# Effect of Extraction Ingredients on the Conformation and Stability of Silk Sericin (SS)

**DOI:** 10.3390/polym14194118

**Published:** 2022-10-01

**Authors:** Munguti Peter Muindi, Ji Hae Lee, HaeYong Kweon, Muo Kasina

**Affiliations:** 1 National Sericulture Research Center, Kenya Agricultural and Livestock Research Organization, Thika 7816-01000, Kenya; 2 National Institute of Agricultural Sciences, Rural Development Administration, Wanju 55365, Korea

**Keywords:** sericin, extraction, sol-gel, viability, proliferation

## Abstract

Silk sericin (SS) has different physicochemical properties depending on the extraction technique. In this study, SS was isolated in the presence of ingredients, including 5 to 10% ethanol (EtOH) and 5 to 10% glycine. Furthermore, temperature conditions of 80 °C, 100 °C, and 120 °C were used for 1, 3, and 5 h to evaluate the extraction rates. The extraction, gelation, structural, and cytotoxicity properties of SS extracted under different conditions were investigated. Extraction at 100 °C and 120 °C were found to have the highest SS yield, with 80 °C being the lowest. SS isolated at 100 °C and 120 °C for 1 and 3 h in water, and EtOH gelled at 4 °C in 2 to 3 days and 37 °C in 40 min. Glycine SS extracts were obtained at 100 °C and 120 °C for 1 h, gelled at 4 °C for 20 days and 37 °C for 16 h. SS was observed at 80 °C, with no gelation occurring. Glycine SS extracts obtained for 3, and 5 h at 120 °C showed no gelation. Circular dichroism (CD) results show glycine in SS induces α-helix and random coil structure. SDS-polyacrylamide gel electrophoresis (SDS-PAGE) and fast performance liquid chromatography (FPLC) were used to quantify the molecular weight distribution at 63 and 70 kDa, respectively. The MMT assay (3-(4,5-dimethylthiazol-2-yl)-2, 5-diphenyltetrazolium bromide) revealed no cytotoxicity in macrophage RAW 264.7 cells treated with this method SS; these findings present the significance and possibility of using selected extraction ingredients in SS that allow for the application of native SS at an initial extraction viscosity.

## 1. Introduction

Scientists have shown an enormous interest in studying silk proteins in recent years, notably silk sericin (SS). SS protein is currently being studied in diverse applications, such as cosmeceuticals, pharmaceuticals, the food industry, cell culture, and biomedical fields [1,2,3]. SS can be molded into a range of forms, including films, sponges, and gels [4]. SS is classified as a boiled water-soluble protein. As a result, in order to use SS-based material, SS must be dissolved in solution form. However, progressive research has pointed out significant impediments to SS application in these fields. One of these critical challenges experienced, especially in the recent SS biomaterial research, is the stability of the extracted SS obtained from the native cocoons. The extraction processes of SS protein are majorly limited to the heating process, famously known as the degumming method [5]. SS obtained by this method quickly transforms from its solution form to gel form when concentration is above 5 wt.% [6]. Gelation of SS can potentially affect the application when a higher viscosity is needed. High SS viscosity increases the rate of SS gelation. The high viscosity is indicative of the high concentration of SS. To slow down the gelation of SS and thus stabilize it in storage for a few hours to days, dilution to lower the concentration can be an option. However, lowering SS aqueous concentration levels poses another challenge where high concentrations might be needed in preparing SS biomaterial [7]. With recent advancements in technology, modifications of the conventional degumming process (boiling) to High-Temperature and High-Pressure (HTHP) have been reported [8]. The HTHP method results in high-quality SS extract with a wide range of molecular weights between 27 and 200 kDa [9]. However, this method does not guarantee SS stability after extraction.

The purpose of this study was to develop a way of stabilizing SS extract for an extended period without changing SS initial concentration viscosity, that is, without diluting the initial extract concentration. That will provide a wide time period before the aqueous SS gels, hence a pliable time for SS use. SS modification has been suggested to stabilize hydrolyzed SS after extraction. The hydroxyl groups in SS have been suggested to play an integral role in the structural conformation of SS protein [10], which has a net effect on the stability of SS. This idea suggests that introducing a stabilization agent that can interact with a hydroxyl group and other functional groups of SS polypeptide during extraction of SS might lead to structural modification and hence the stability of SS. Numerous studies have described the stability and conformation of SS after dissolution using circular dichroism (CD), a powerful tool for characterizing the secondary structure and conformation of aqueous proteins [11,12,13,14]. As a result, we investigated the structural properties of SS solution, semi-gel, and gel. Using extraction ingredients in this method is a novelty that has not been fully elucidated and therefore lays the foundation to further research on understanding the utilization of different extraction ingredients in SS stabilization.

## 2. Experimental Section

### 2.1. Material and Methods 

Silkworm cocoons were obtained from the National Institute of Agricultural Sciences (NAS), Rural Development Administration, Korea, and reared in a controlled environment.

### 2.2. SS Extraction in an Autoclave Using Pure Water

Following the protocol published [8] with some modifications, *Bombyx mori* cocoons were cut to remove the pupa; they were then cut into small flakes to increase the surface contact with the extraction solvent. SS was extracted by a degumming method using an autoclave at 80, 100, and 120 °C (1 kgf/cm^2^) for 1, 3, and 5 h, respectively. Extraction bath ratio of 1:20 (g/mL) was used. The extracted SS was filtrated at 50 °C in a quadruple-layered Mira cloth (Millipore, Burlington, MA, USA) with a pore size of 22–25 µm to separate SS from fibroin and eliminate impurities that might be present in the solution. The SS was cooled to room temperature, and SDS-PAGE was used to estimate the molecular weight of the filtrate.

### 2.3. Preparation of EtOH Non-Precipitated SS

SS extracted using low concentrated ethyl alcohol (EtOH, Sigma-Aldrich, St. Louis, MO, USA ) was prepared using a previously described method [8,15] with some modifications. The cocoons were cut and prepared as described above. SS was extracted by autoclaving the silkworm cocoons with low-concentration EtOH at 5% and 10% at 80, 100, and 120 °C (1 kgf/cm^2^) for 1, 3, and 5 h, respectively. The filtration parameters and cooling conditions refer to procedure 2.2.

### 2.4. Preparation of Glycine Non-Precipitated SS

The cocoons were cut and prepared as described above [8]. SS was autoclaved in a bath ratio of 1:20 g/mL of glycine (Sigma-Aldrich, USA) 5 and 10 *w/v*% at 80, 100, and 120 °C (1 kgf/cm^2^) for 1, 3, and 5 h, respectively. The filtration parameters and cooling conditions refer to procedure 2.2.

### 2.5. Characterization of SS Filtrate

#### 2.5.1. Molecular Weight Estimation of SS Filtrate with SDS-PAGE

To the aliquot of the initial SS extract, 3 µL of the sample was added to 22 µL 8M urea (Sigma), then 10 µL SDS-PAGE sample (4 × buffer, Bio-Rad Laboratories, Hercules, CA, USA) was added and heated at 50 °C for 30 min, for complete decomposition of SS protein. SDS-polyacrylamide gel electrophoresis (SDS PAGE) was performed according to the standard protocol of Laemmle [4] by using a readymade 4–15% gradient gel (Bio-Rad Laboratories, USA.). After the electrophoresis, the gel was silver stained with a silver staining kit (Invitrogen, Waltham MA, USA). The molecular weight of the SS protein was estimated using precision protein standards (Bio-Rad Laboratories, USA)

#### 2.5.2. Fast Performance Liquid Chromatography (FPLC)

**Figure 1:**
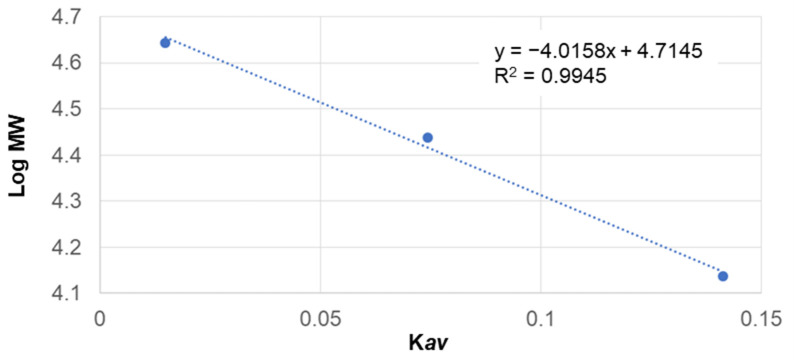
Gel Filtration Kit Calibration Low MW.

The molecular weight distribution of SS extracts from pure water, EtOH, and glycine was performed with FPLC by Superdex™ 30 increase 10/300 GL column (Sigma-Aldrich, USA). Standard Gel Filtration Calibration Kit LMW (Cytiva, Marlborough, MA, USA). 0.1 wt. % sample was membrane filtered (0.2 µL); a 7 mL filtrate sample was injected. The peptides were separated using a column flow rate of 0.5 mL/min and detected at wavelengths of 215 nm. The calibration curve was plotted using the gel-phase distribution coefficient (*Kav*) versus the logarithm of the molecular weight Equation (1), and the standard curve (Figure 1).

(1) $$Kav=\frac{Ve-Vo}{Vc-Vo}$$

*Kav* is a function of the elution volume of a molecule where: *Ve* is the elution Volume, *Vo* Column Void Volume, and *Vc* is the Geometric Column Volume.

#### 2.5.3. Circular Dichroism (CD) of SS Solution

After extraction, the CD evaluated SS conformation changes in solution and gel forms. The spectrum was recorded by a JASCO J-1500 CD Spectrometer (Oklahoma City, OK, USA) with a wavelength range of 190–260 nm. The SS solution was adjusted to a concentration of 0.01 wt. %. In the case of SS gel, the gel was observed in its native state without dilution. The smearing method was done to the gel in a quartz cell with a 0.1 mm path length and an accumulation of 3 scans.

#### 2.5.4. SS Gelation Monitoring

SS gelation was determined at two different temperature conditions, at 4 °C and 37 °C. On separate conditions of 4 °C and 37 °C, an aliquot of 1.5 ml of SS solution was pipetted into a vial to follow the gelation process and to determine gelation time. The sol gel transition was evaluated by the tilting method each day per vial. The gelation time was defined as the period from the first day to when the solution became viscous, and there was no flow upon inverting the vertically positioned vial. SS gelation was also observed at 37 °C. And gelation was determined when SS became viscous, and there was no flow when the vertically positioned vial was inverted.

#### 2.5.5. Fourier Transformed Infrared (FT-IR) Analysis 

The SS solution and gel were cast into a thin film and dried for the FT-IR analysis (PerkinElmer Spectrum 100 Series, Waltham, MA, USA). To confirm the possible structural changes of SS, the backgrounds and spectral scans were set in the wavelength range from 4000 to 400 cm^−1^, at a resolution of 4 cm^−1^, in 32 scans.

#### 2.5.6. Cytotoxicity Assay of SS Solution

Raw 264.7 cell line was cultured in DMEM, containing 10% fetal bovine serum and 1% antibiotic at 37 °C with 5% CO_2_. When the cells reached 90 % confluence, the well was sub-cultured and incubated at 37 °C. The cell viability was measured using kit (EZ-Cytox, Do-Gen Bio, Seoul, Korea). RAW 264.7 cells (3 × 10^4^ cells/mL in 200 µL culture medium) were seeded into 96-well plates at 37 °C for 24 h before treating the cell. The RAW 264.7 cell was exposed to various concentrations (1–1000 µg/mL) of each type of SS for 24 h at 37 °C. EZ-Cytox (100 ×) was added to test the cell viability, followed by incubation for 1 h. The absorbance was measured at 450 nm using a Multiskan™ Go Microplate spectrophotometer (Thermo Fisher Scientific, Waltham, MA, USA). 

#### 2.5.7. Raw 264.7 Cell Proliferation after SS Solution and Gel Treatment

The cell proliferation of RAW 264.7 was investigated every 24 h for 4 days. Briefly, the cells were inoculated into 6-well plates at a density of 3 × 10^4^ cells/mL in a 2 mL culture medium for 24 h. The RAW 264.7 cell was exposed to various concentrations (1–1000 µg/mL) of each type of SS for 24 h at 37 °C, and cells were counted using LUNA-FL Dual Fluorescence Cell Counter (Logos Biosystems, Anyang, Korea).

## 3. Results and Discussion

### 3.1. SS Concentration (wt. %)

Table 1 shows the SS extract concentration ratio obtained by the HTHP method at different temperatures of 80, 100, and 120 °C and times of 1, 3, and 5 h, respectively. The extraction concentration of SS obtained at 80 °C was lower than that obtained at 100 °C and 120 °C with the amount of SS in the cocoon, as previously reported by Zhang [16]. However, the extraction concentration of SS was found to increase with the extraction ingredient. Some external stimuli, such as temperature and solvents, can trigger the interaction of polar and non-polar molecules of SS, hence increasing dissolution rates.

### 3.2. The Molecular Weight Distribution of Degummed SS Protein

**Figure 2:**
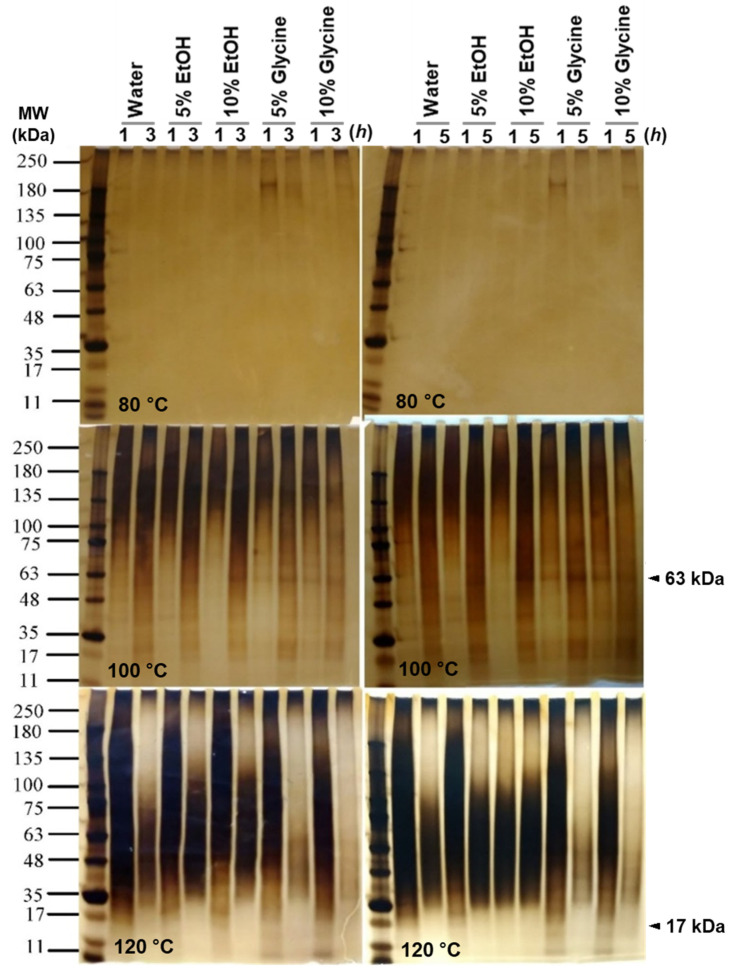
SDS-PAGE of SS protein under various extracting conditions. SS was extracted with various ingredients (water, 5–10% EtOH, and 5–10% glycine), temperature (80–120 °C) and time (1–5 h). A 4–20% gradient SDS-PAGE gel (Bio-Rad) was used.

The temperature and duration of extraction substantially impact SS molecular weight (MW) [9]. SDS-PAGE is a helpful approach for determining MW [17]. The approximate molecular weight distribution of SS obtained in pure water, Glycine and EtOH are shown (Figure 2). At 80 °C extraction condition, only SS extracted in glycine showed a clear polypeptide band at 180 kDa; there was no indefinable protein band pattern in water and EtOH SS extract at this temperature.

The smeary band patterns of SS extract in pure water and EtOH at 100 °C exhibits no distinctive band pattern. On the other hand, SS extract in glycine reveals a distinct and apparent band pattern at 63 kDa for SS extracted in 1, 3, and 5 h, respectively. At 120 °C, SS extract from water and EtOH showed no distinctive band pattern. However, SS extract in glycine shows a distinct protein band of approximately 17 kDa for a one-hour extraction condition. The glycine 5% extract has a more noticeable and stronger band than the glycine 10% extract.

**Figure 3:**
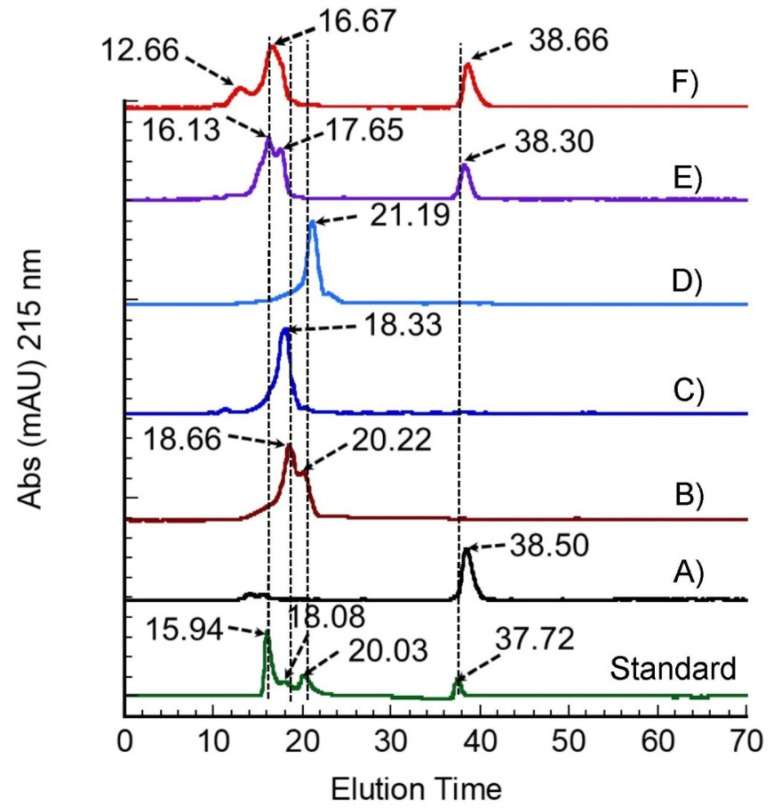
Fast performance liquid chromatography of SS extracts; (**A**) Pure Glycine, (**B**) water, (**C**) EtOH 5%, (**D**) EtOH 10 %, (**E**) Glycine 5 %, (**F**) Glycine 10 % and MW Standard.

Takasu et al. reported the SS protein is composed of three major polypeptides with molecular weights of 400, 250, and 150 kDa [18]. Kweon et al. reported 5 bands from 250 to 40 kDa [3]. Sprague et al.; reported nearly 15 different SS bands, ranging from about 20 to 220 kDa [19]. SDS-PAGE to determine molecular weight might not provide the correct expected molecular weight. A macromolecule can be post-transnationally modified by glycosylation, increasing its mass. From these results, SS extracted at 100 °C for one hour in all the “conditions” appeared to have been partially hydrolyzed and was different in molecular weight distribution compared to the product extracted at 120 °C. To quantitatively determine the molecular weight, FPLC was performed using the samples from 100 °C for one hour (Figure 3). Table 2 shows SS molecular weight distribution. Most peaks were found between 16–21 min, which indicates 60 to 69 kDa. Glycine-added sericin showed peaks at 38 min, that same retention time with pure glycine.

### 3.3. Secondary Structure of SS Solution and Gel

**Figure 4:**
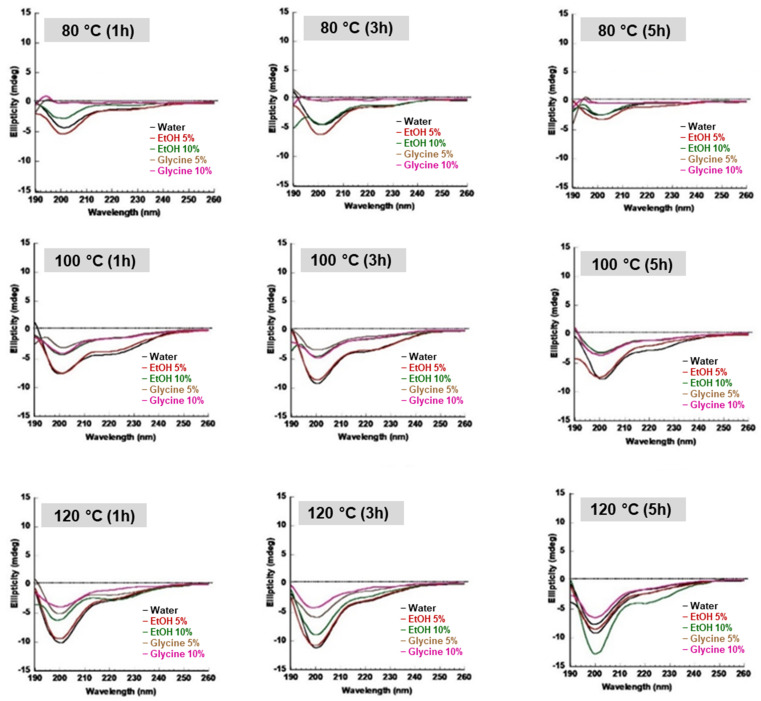
CD spectrum of SS solution. The secondary structure of SS was investigated according to extracted temperature (80 °C, 100 °C, and 120 °C) and time (1, 3, and 5 h).

**Figure 5:**
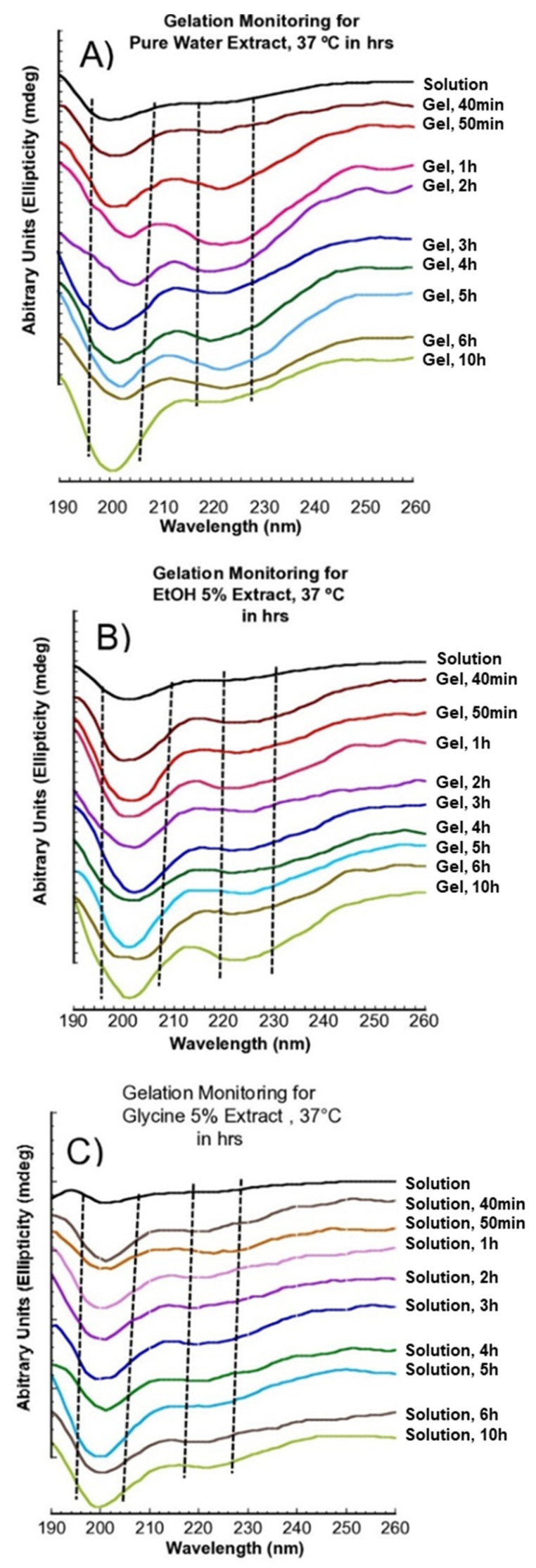
CD spectra of SS with time. (**A**) Gelation of water extract at 37 °C; (**B**) gelation of EtOH 5% at 37 °C; (**C**) gelation at glycine 5% at 37 °C.

**Figure 6:**
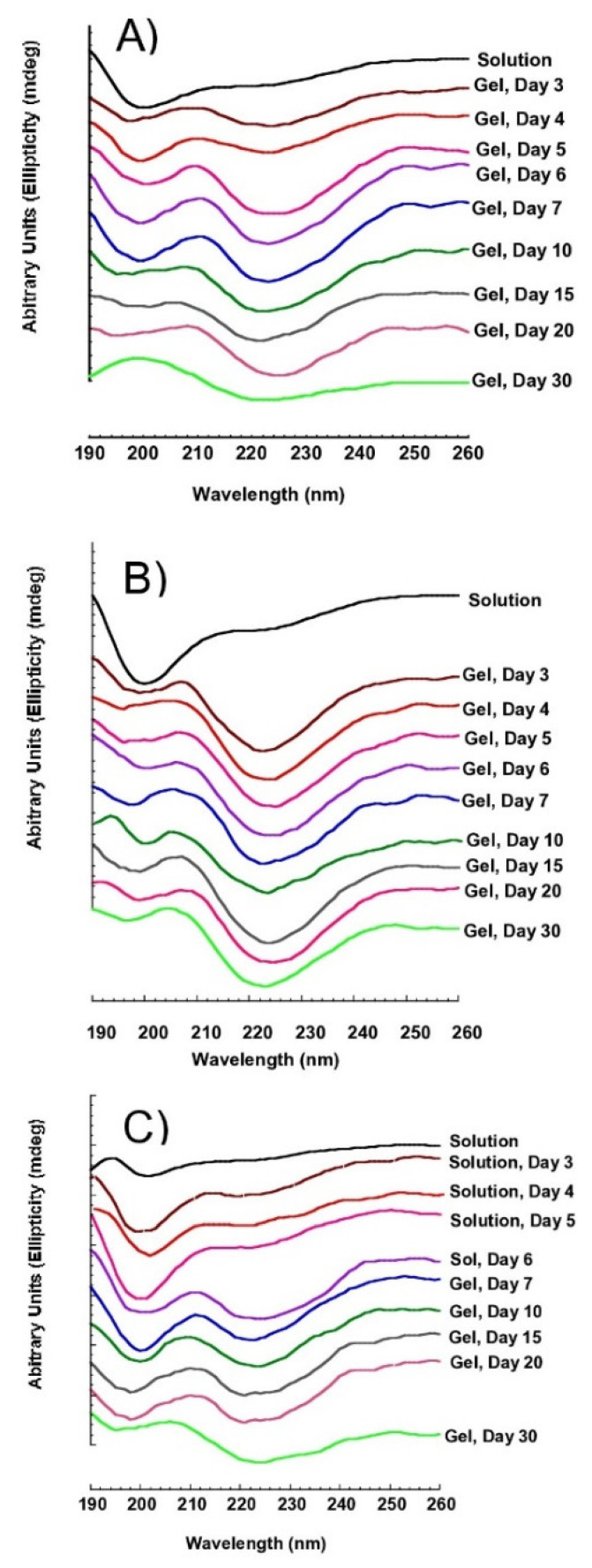
CD spectrum of SS Gel at 4 °C. (**A**) Water SS extract, (**B**) EtOH 5% SS extract, and (**C**) glycine 5% SS extract.

Circular Dichroism (CD) is a powerful and effective method for studying the secondary structure conformation of proteins [14,15]. Protein conformation is induced by several factors, such as temperature, pH, and solvent [20]. Figure 4 shows SS aqueous secondary structural conformation obtained under different extraction ingredients. At 80 °C, SS extract in water, EtOH 5%, and 10% show a negative peak around 198 nm and a shoulder peak around 218 nm attributed to the random coil and β-sheet [14], for 1, 3, and 5 h conditions, respectively. SS extracts from glycine (5% and 10%), at 80 °C for 1, 3, and 5 h were observed to have a positive peak around 192–193 nm, attributed to α-helix conformation [21]. At 100 °C and 120 °C for 1, 3, and 5 h, SS extract with glycine was observed to conform to the random coil and β-sheet orientations with a broad negative peak around 197–202 nm.SS extracted at 100 °C for one hour was observed to have a broad and high molecular weight distribution in the SDS-PAGE (Figure 2) and FPLC (Figure 3). To study SS gel secondary structure, arbitrarily selected SS extract was obtained in pure water, EtOH 5%, and glycine 5% for one hour at 100 °C. SS gel transition was studied under two conditions, 37 °C and 4 °C, as observed in Figure 5 and Figure 6, respectively. SS water extract gelled in 40 min at 37 °C, as shown in Figure 5A. The transition of SS solution to gel was detected with a shoulder peak around 218 nm, attributed to the β-sheet [14] structure. To study the aqueous phase, the structural transition of SS in CD, the spectrum was observed as a random coil, Figure 4. The peak intensity around 218 nm was visualized to increase over time. However, the transition was detected with a lower peak in this location after 10 h. Temperature and time induce this occurrence. Figure 5B displays SS extract in EtOH 5%. At 37 °C, SS extract from EtOH 5% gelled within 40 min.

In the CD, the SS solution observed a random coil peak at 200 nm, and a shoulder peak around 218 nm attributed to β-sheet [14]. The peak intensity in EtOH 5% extract around 218 to 220 nm was observed to intensify with time, suggesting a SS effect in the EtOH environment. In Figure 5C, SS obtained in glycine 5%, visualized in the CD with a positive peak around 192–193 nm, attributed to α-helix structure and a negative peak around 200 nm attributed to the random coil. Glycine is known as a secondary structure disruptor and is affected by serine, threonine, tryptophan, and tyrosine residues which are building blocks of natural SS [22]. Therefore, glycine addition may change the structural properties of SS. Glycine SS extract was observed to form a gel after 16 h. The gel observed in the CD with negative peaks at 198 to 202 nm, and a shoulder peak around 218 to 220 nm was attributed to the random coil and β-sheet structure. The peak around 218–220 nm was observed to intensify after 18 h; these indicated SS glycine extract remained randomly stable for 16 h.

The SS structural transition in days Figure 6 was studied at 4 °C, which is the general storage condition for SS; the structural change was observed to intensify in all the samples over time from random coil to the β-sheet structures. The transformation of the random coil peak at 198 to 200 nm was observed to decrease, while the β-sheet peak around 218–220 nm was observed to increase. Interestingly, Figure 6C shows that the glycine extract transition was slower compared to water Figure 6A and EtOH 5% Figure 6B extracts.

### 3.4. Stability Monitoring of SS Aqueous at 4 °C

**Figure 7:**
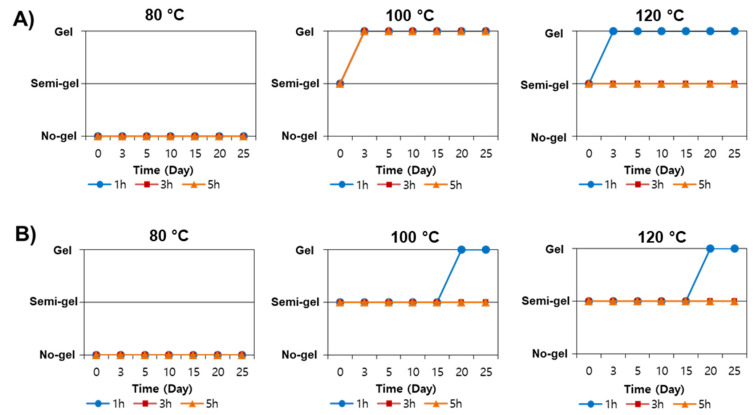
Gelation time of SS at 4 °C with different extraction conditions. (**A**) The gelation status of SS was obtained from water and 5–10% EtOH. (**B**) The gelation status of SS was obtained from 5–10% glycine.

Phase stability is important to fabricate SS for various applications, including in pharmaceuticals and biomedical fields. SS has been reported to gel within an hour after extraction when the concentration is above 5% (*w/v*) [6]. Once the aqueous phase transforms into a gel, it makes it difficult to fabricate SS. In this study, the stability of SS was monitored at 4 °C for 1, 3, and 5 h of extraction, as shown in Figure 7. SS solution extracted at 80 °C did not form a gel due to low SS concentration levels (Table 1). It was observed that SS extracted in pure water, EtOH 5% and 10% gelled quickly within 3 to 4 days at 4 °C storage, as shown in Figure 7A. SS obtained in pure water, and EtOH 5% and 10% at 100 °C for 1, 3, and 5 h and 120 °C for 1 h were observed to form a gel. On the other hand, gelation did not occur for SS extracted for 3 and 5 h at 120 °C. SS obtained in glycine forms a gel at most on the nineteenth to twentieth day. Even more intriguing, only SS obtained in 1 h under 100 °C and at 120 °C gelled. Contrary, at 100 °C and 120 °C, gelation was not observed between 3 to 5 h. In a previous study, α-helix structure reduced the gelation properties compared to β-sheet [23]. Increases in α-helix structure in glycine-mediated SS extract may delay the gelation process.

### 3.5. SS Gel Thermal-Reversibility

**Figure 8:**
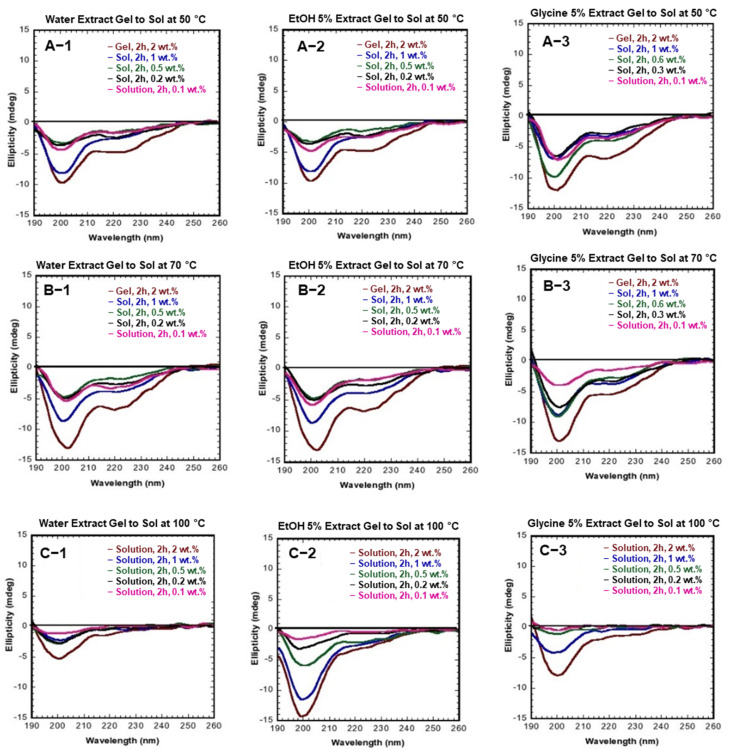
Gel to sol structural conformational transition. (**A-1**–**A-3**) at 50 °C, 2 h; (**B-1**–**B-3**) at 70 °C, 2 h; (**C-1**–**C-3**) at 100 °C, 2 h.

**Figure 9:**
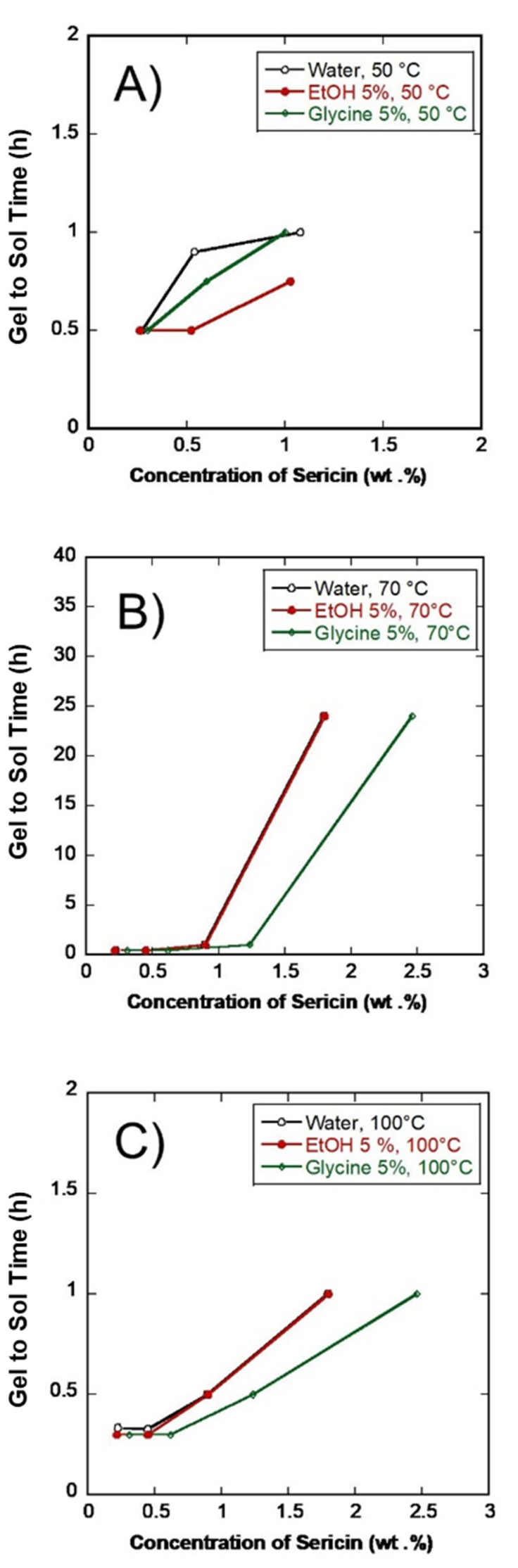
Gel to sol transition at different temperatures. (**A**) At 50 °C; (**B**) at 70 °C; (**C**) at 100 °C.

The structural transformation of SS random coil to β-sheet [24] is known to occur due to its innate sticky-like characteristics that generate intermolecular hydrogen bond interactions [7]. SS is thermally reversible at high temperatures and re-gels when cooled [6]. The SS gel to sol transition is shown in the CD spectrum in Figure 8 at various temperatures. Figure 8 (A-1, A-2, and A-3) represent water, EtOH 5%, and Glycine 5%, gel transition at 50 °C, respectively. In this condition, SS gel formed from the dilution, i.e., 1, 0.5, and 0.2 wt. % were transformed from sol after 2 h Figure 9A. A lower concentration of 0.1 wt.% did not form a gel. A decrease in SS concentration means lowering its viscosity, which leads to a delay in SS gelation [25]. The initial concentration of 2.wt.% did not develop any sol for two hours, even after 24 h. (Data not presented here). At 70 °C, the CD spectrum shows water, EtOH 5%, and Glycine 5% gel transition Figure 8 (B-1, B-2, and B-3). At this temperature, the transition of SS gel to sol was similar to that of 50 °C conditions, though the 2 wt.% gel becomes sol after 24 h, as shown in Figure 9B. The CD spectrum reveals a prominent peak at 218 nm at 2 wt.%, linked to the β-sheet structure. The gel transitions at 100 °C for water extract, EtOH 5%, and Glycine 5% are shown in Figure 8 (C-1, C-2, and C-3, respectively). SS gel to sol was observed in all concentrations Figure 9C, and in the CD, a peak of around 200 nm was due to a random coil.

### 3.6. IR Spectroscopy of SS Solution and Gel

**Figure 10:**
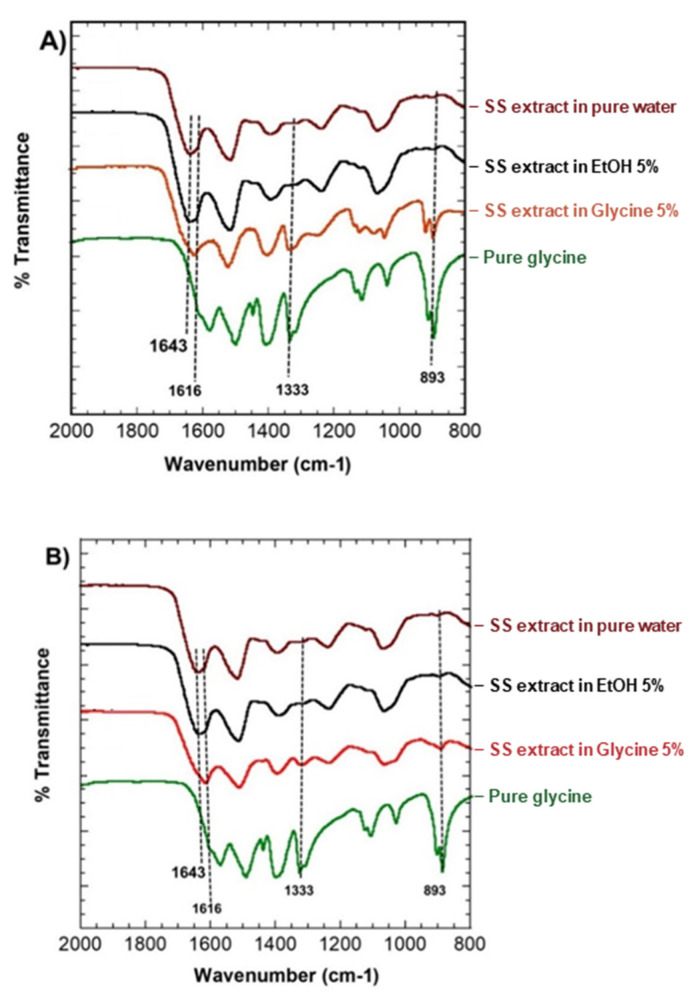
IR spectroscopy of (**A**) SS solution film (**B**) Gel film dried at 60 °C. The samples were SS extract in pure water, EtOH 5%, glycine 5%, and pure glycine spectrum, respectively.

The secondary structures of SS solution and the gel were compared in the FT-IR spectra of cast film at 60 °C, as shown in Figure 10. In most cases, the amide I spectrum serves as a standard reference for the protein secondary structure [26]. In Figure 10, the spectra of the solution and the gel are comparable (A and B). Some structural changes are more apparent, especially in films derived from glycine SS. The random coil and intermolecular β-sheet structure are attributed to the sharp peak at 1643 cm^−1^ and the shoulder peak at 1616 cm^−1^, respectively [27,28]. This indicates that SS solution dried to a film at 60 °C has a high degree of a random coil and a shoulder peak of β-sheet crystallization at this point. SS water gel film with a peak at 1643 cm^−1^, shown in Figure 10B. This peak indicates that SS intermolecular interactions generate a more stable structure during gelation than solution film. Difference peaks between the 5% EtOH solution and gel film were observed due to the random coil and intermolecular β-sheet conformations, a broad peak identical to the Amide 1 region area could be detected at 1643 cm^−1^, as well as a shoulder peak at 1616 cm^−1^. This means that non-precipitating EtOH stabilized the SS solution and gel film structure. Glycine 5% SS solution and gel films were also compared. β-sheet was identified as a sharp weak peak in the amide I at 1616 cm^−1^. However, in the gelation assay, glycine extract reveals a delayed timeframe of exhibiting higher levels of stability (Figure 7). This shows that a weak and sharp band on the films could be due to glycine residue on the surface, with comparable spectra; these two glycine peaks were also observed in 1333 and 893 cm^−1^. The NH_2_ twist + CH_2_ twist are ascribed to these peaks. [29].

### 3.7. Cell Viability and Cell Proliferation

**Figure 11:**
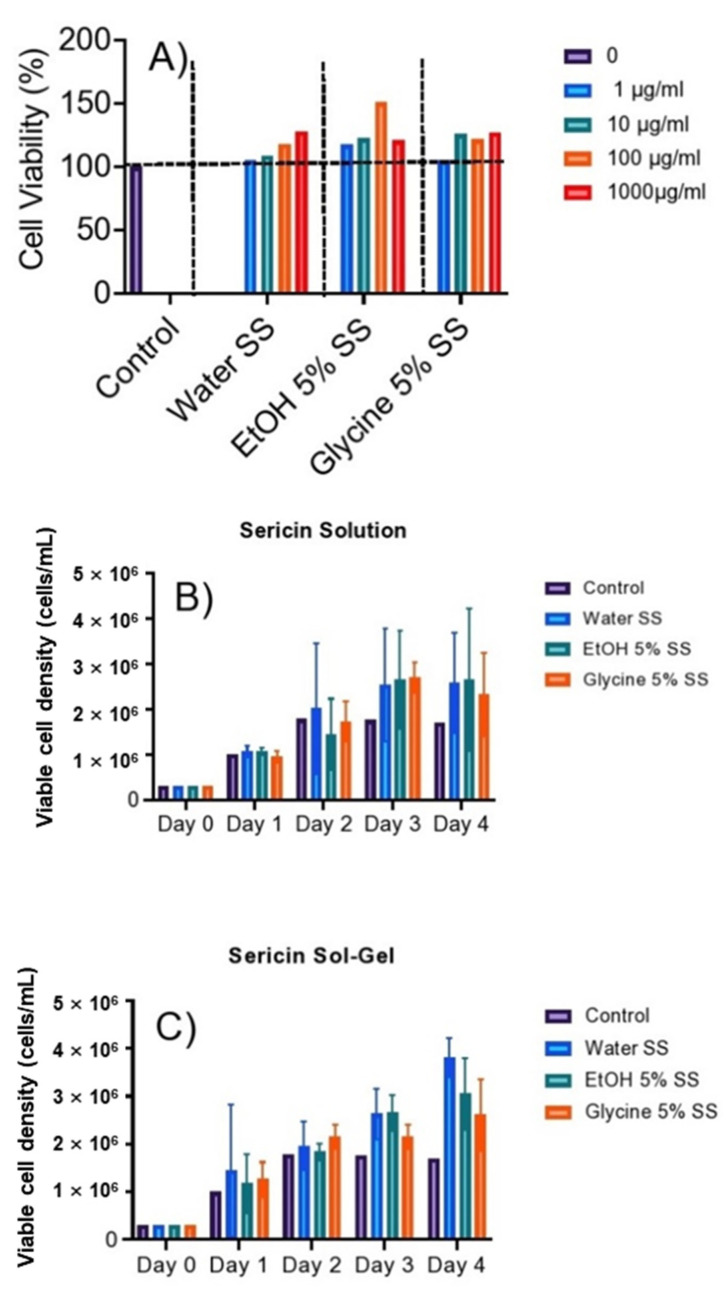
Viability and proliferation of SS affect SS protein. (**A**) Cytotoxicity of SS extract on RAW 264.7 cells using various extraction ingredients. (**B**) RAW 264.7 cell growth in SS solution derived from various stabilizers. (**C**) RAW 264.7 cell proliferation in SS gels derived from various stabilizer agents.

RAW 264.7 cells were treated with SS solution at various concentrations (1 to 1000 µg/mL) and incubated for 24 h (Figure 11A). SS extract from water, 5% EtOH, and 5% glycine treatment to RAW 264.7 cells show more than 90% cell viability; these results suggest that SS obtained using water, EtOH, and Glycine does not induce cell apoptosis and substantially enhanced RAW 264.7 cytokinesis for cells treated with SS solution and gel (Figure 11B) and sol gel (Figure 11C) respectively. The natural cell cycle progression causes the intensity of cells to fluctuate from day one to day four. The overall results suggest that SS promote cellular viability and induce cell intensity (proliferation) in all the extraction condition.

## 4. Conclusions

We compared SS protein extraction to water, EtOH, and glycine. The results revealed that glycine extraction yielded/concentration the highest extraction percentage of SS protein content, followed by ethanol than water, respectively. The method of SS extraction had a significant impact on SS stability. The tilting method, turbidity change, and CD spectroscopy were used to investigate the stability behaviour of SS. Water and EtOH SS extracts had very low stability in the general storage condition at 4 and 37 °C, whereas Glycine extract had high stability in both conditions. The SDS-PAGE and Fast Performance Liquid Chromatography (FPLC) were used to evaluate the molecular weight distribution. The effects of SS extract on cell viability were significant. The MTT assay revealed that the SS solution and gel from all extractions were not toxic to RAW 264.7 cells. Cell viability was observed to increase at certain concentrations with no toxicity. When SS solution and gel were injected or treated with RAW 264.7 cells, there was a slight difference in cell viability and cell viability intensity.

## Figures and Tables

**Table 1:** Comparison of SS extraction concentration (wt.%) by various extraction ingredients, temperature, and time.

Extraction Ingredients	Time (h)	Temperature (°C)		
		80 °C	100 °C	120 °C
Water	**1**	0.06	0.40	1.63
	**3**	0.09	0.64	1.88
	**5**	0.09	1.00	1.98
EtOH 5%	**1**	0.08	0.20	1.32
	**3**	0.09	0.70	1.70
	**5**	0.09	0.74	1.86
EtOH 10%	**1**	0.07	0.90	1.23
	**3**	0.16	1.00	1.74
	**5**	0.22	1.23	1.86
Glycine 5%	**1**	0.03	0.75	1.60
	**3**	0.04	1.03	1.70
	**5**	0.04	1.60	2.10
Glycine 10%	**1**	0.03	0.80	1.61
	**3**	0.04	1.03	1.86
	**5**	0.05	1.61	2.01

**Table 2:** Molecular weight distribution in FPLC.

Parameter	Retention Time (min)	*Kav*	Log MW	MW (kDa)
Pure glycine	38.50	-	-	-
Water extract	18.66	0.000681	1.173818	69.5
	20.22	0.005014	1.172739	69
EtOH 5% extract	18.33	0.000872	1.173771	69.5
EtOH 10% extract	21.19	0.004086	1.172970	69.2
Glycine 5% extract	16.13	0.102703	1.148413	60
	17.65	0.033747	1.165584	65.65
	38.30	-	-	-
Glycine 10% extract	12.66	0.006551	1.172356	69
	16.67	0.001366	1.17348	69.5
	38.66	-	-	-
